# Chaotic Extension Neural Network Theory-Based XXY Stage Collision Fault Detection Using a Single Accelerometer Sensor

**DOI:** 10.3390/s141121549

**Published:** 2014-11-14

**Authors:** Chin-Tsung Hsieh, Her-Terng Yau, Shang-Yi Wu, Huo-Cheng Lin

**Affiliations:** Department of Electrical Engineering, National Chin-Yi University of Technology, Taichung 41170, Taiwan; E-Mails: fred@ncut.edu.tw (C.-T.H.); estyeon@gmail.com (S.-Y.W.); aaa15503602@gmail.com (H.-C.L.)

**Keywords:** master and slave chaos error systems, extension neural network, XXY stage, dSPACE

## Abstract

The collision fault detection of a XXY stage is proposed for the first time in this paper. The stage characteristic signals are extracted and imported into the master and slave chaos error systems by signal filtering from the vibratory magnitude of the stage. The trajectory diagram is made from the chaos synchronization dynamic error signals *E*_1_ and *E*_2_. The distance between characteristic positive and negative centers of gravity, as well as the maximum and minimum distances of trajectory diagram, are captured as the characteristics of fault recognition by observing the variation in various signal trajectory diagrams. The matter-element model of normal status and collision status is built by an extension neural network. The correlation grade of various fault statuses of the XXY stage was calculated for diagnosis. The dSPACE is used for real-time analysis of stage fault status with an accelerometer sensor. Three stage fault statuses are detected in this study, including normal status, Y collision fault and X collision fault. It is shown that the scheme can have at least 75% diagnosis rate for collision faults of the XXY stage. As a result, the fault diagnosis system can be implemented using just one sensor, and consequently the hardware cost is significantly reduced.

## 1. Introduction

**Figure 1.**
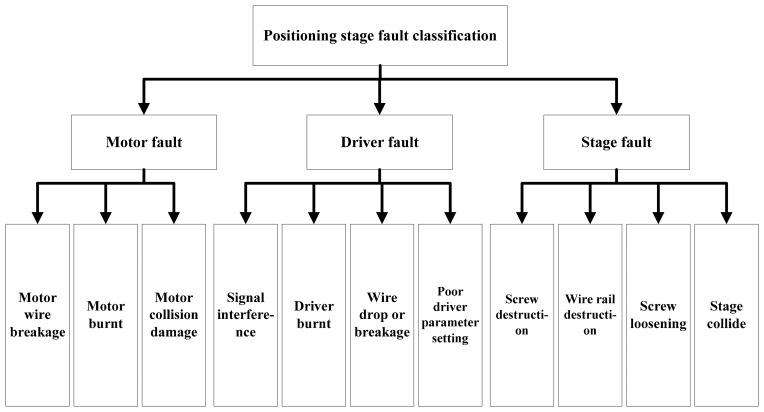
Positioning stage fault classification and possible causes.

Automatic production has become a common industrial practice to achieve efficiency and cost savings. The components requiring precision operation in the automatic system needs a good positioning system as support, in order to achieve uniformity in the products. Hence, a positioning stage can avoid low product yields that would lead to increased cost [1,2]. There are multiple positioning stage options, such as the traditional XYθ stage, the improved XXY stage, the UVW stage and other special stages providing good positioning functions. The stage used in this study is a XXY stage, which not only provides high accuracy positioning, but also remedies the defects and problems in traditional stages with its special single-layer design, thus achieving good weight capacity [3]. In the automatic process, even machinery with the highest precision will encounter faults during operation, which may be caused by improper maintenance of the positioning stage or human negligence in operation. There are few studies focusing on the control or design of the positioning stage, and discussions of positioning stage faults are also rare. There is a lack of a good fault detection system to effectively detect faults. These problems are addressed in this paper. The possible causes of the positioning stage fault classification are shown in Figure 1. The XXY positioning stage fault classification diagram was provided by Chiuan Yan Technology Co., Ltd. (Changhua, Taiwan). As seen, the major faults can be divided into motor, driver and stage. This study focused on the XXY collision faults.

In normal operation, when there is wrong movement in motor operation due to human negligence or the internal calculation exceeding the machine movement limit, the sliding stage would move beyond the moving range. At this point, the machine triggers the photosensor as a protector, meaning that the positive or negative limit point of the machine is about to be reached. Thus, the stage is stopped forcibly to prevent damages. However, when the stage movement is about to exceed the limit range and the protector is not triggered, the motor continues running, and the components collide with each other, thus damaging the stage. This is called collision fault of the stage. Collision faults cause related stage damage. The impact between stage parts leads to abrasion, even destruction, and the stage has errors in positioning that causes positioning errors. In terms of motor, the motor may be damaged, even burnt. As for the stage, where the stage components collide they may suffer displacements or deformations, thus lowering the positioning accuracy. Some effects of stage collisions on the stage may not be visible, thus, it is important to identify whether the stage has a collision fault when the stage is moving. Otherwise, the collision fault does not only occur in the XXY stage of this study, but also may exist in other two or more dimensional sliding stages. The proposed method could be applied to other platforms for collision fault diagnosis.

**Figure 2.**
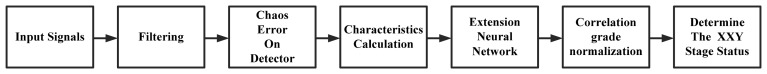
Diagnostic flow chart for XXY stage.

In order to detect the stage collision status, the vibration signal naturally derived from the positioning stage is used as the fault feature. The installation of sensors is reduced by single signal acquisition. In terms of detection methods, there are many methods with characteristic signal analysis at present. The complete signal analysis methods include spectral analysis [4–7], wavelet analysis [8–12] and neural network [13–16]. This study used an accelerometer to measure the vibration signala on the XXY positioning stage. The characteristic signal was extracted by filtering. The extracted signal passed through the master and slave chaotic systems. The signal characteristic was strengthened by using chaotic characteristics. The original one-dimensional signal was changed into a chaotic dynamic error to become a three-dimensional signal. The trajectory diagram was plotted based on this dynamic error. The pattern characteristics were extracted from the error trajectory diagram. The matter-element model was built by the pattern characteristics and extension neural network theory, so as to determine the positioning stage fault statuses. The signals extracted from the stage were used for spectral analysis, and the signals were extracted from the analysis result. These signals were extracted by vibration signals. Therefore, the signals were too small to review the stage status, and the extracted signals were imported into different master and slave chaotic signal systems. The system dynamic error *E* was extracted. The dynamic error trajectory diagram derived from the characteristic signal was drawn. The characteristic pattern variation of the various statuses was selected. Finally, the distance and maximum and minimum distances between positive and negative centers of gravity of pattern characteristics were used as the pattern characteristics for stage status recognition. The matter-element model most similar to the analyte was calculated by using the extension set for identification. The larger calculated value of the correlation function was more similar to the status, and the maximum value was the stage status. Finally, the dSPACE was used for the required real-time status monitoring. The diagnostic flow chart of this study is shown in Figure 2.

## 2. Master and Slave Chaotic Systems

The master and slave chaotic systems are built based on the chaos theory. Chaos theory [17] is a nonlinear system theory. The signal derived from the chaos system generates an orderly but non-periodic kinematic trajectory due to the chaotic attractor. This kinematic trajectory changes significantly due to minor signal changes. The naturally pursued dynamic error in the two chaotic systems is extracted by the characteristics of the master and slave chaotic systems used in this study. Therefore, the dispersion between the master and slave chaotic systems should be obtained in the calculation. The value is the dynamic error of system. Taking the Lorenz system as an example, the Lorenz chaos system is a chaotic system proposed when Lorenz proposed the chaos theory, the nonlinear differential equation system expression is shown as follows, where *x*, *y* and *z* are status variables, *a*, *b* and *c* are system parameters:

(1) $$\begin{array}{l} \frac{dx}{dt}=a(y-x)\\ \frac{dy}{dt}=x(b-z)-y\\ \frac{dz}{dt}=xy-cz \end{array}$$

Lorenz master-slave system is constructed as follows:Master System:

(2) $$\{\begin{array}{l} \frac{dx_{1}}{dt}=a(x_{2}-x_{1})\\ \frac{dx_{2}}{dt}=bx_{1}-x_{1}x_{3}-x_{2}\\ \frac{dx_{3}}{dt}=x_{1}x_{2}-cx_{3} \end{array}$$

Slave System:

(3) $$\{\begin{array}{l} \frac{dy_{1}}{dt}=a(y_{2}-y_{1})+u_{1}\\ \frac{dy_{2}}{dt}=by_{1}-y_{1}y_{3}-y_{2}+u_{2}\\ \frac{dy_{3}}{dt}=y_{1}y_{2}-cy_{3}+u_{3} \end{array}$$

where *u*_1_, *u*_2_ and *u*_3_ are the controls of slave system. The normal XXY stage signal is added in the master system to form a discrete signal S_1_[*n*], and the variables are defined as *x*_1_ = S_1_[*i*], *x*_2_ = S_1_[*i* + 1] and *x*_3_ = S_1_[*i* + 2], where *i* = 1, 2, 3,‥, n − 2 forming the sampled data series bearing fault signal. The fault XXY stage signal is added in the slave system to form a discrete signal S_2_[*n*], where the variables are defined as *y*_1_ = S_2_[*i*], *y*_2_ = S_2_[*i* + 1] and *y*_3_ = S_2_[*i* + 2]; S_2_ represents the sampled data series bearing the normal signal, *n* represents the total number of sampled data in a complete cycle, so the master-slave system error status can be expressed as *e*_1_ = *x*_1_ − *y*_1_, *e*_2_ = *x*_2_ − *y*_2_, *e*_3_ = *x*_3_ − *y*_3_, and the dynamic error system (ED) is changed to:

(4) $$ED:\{\begin{array}{l} \frac{de_{1}}{dt}=E_{1}=a(e_{2}-e_{1})-u_{1}\\ \frac{de_{2}}{dt}=E_{2}=be_{1}-e_{2}-e_{1}e_{2}-y_{1}e_{3}+y_{3}e_{1}-u_{2}\\ \frac{de_{3}}{dt}=E_{3}=e_{1}e_{2}+y_{1}e_{2}+y_{2}e_{1}-ce_{3}-u_{3} \end{array}$$

According to [17], when there is no control input, the system parameters *a*, *b* and *c* must meet Equation (5), then the system has a chaotic attractor:

(5) $$\begin{array}{l} \lambda_{1}=-\frac{a+1}{2}+\frac{1}{2}\sqrt{{\left(a+1\right)}^{2}+4a(b-1)}\\ \lambda_{2}=-\frac{a+1}{2}-\frac{1}{2}\sqrt{{\left(a+1\right)}^{2}+4a(b-1)}\\ \lambda_{3}=-c \end{array}$$

The convergence rate of chaos dynamic error can be controlled by *u*_1_, *u*_2_ and *u*_3_, and the important characteristics of different fault behaviors are formed by using the dynamic error trajectory diagram of *E*_1_, *E*_2_ and *E*_3_ on the phase plane. The signals extracted by the positioning stage are filtered, and these signals are added as analytic signals into the slave system, so as to obtain the dynamic error signal for the master system. Finally, the dynamic error trajectory is drawn according to the dynamic errors *E*_1_ and *E*_2_. The trajectory patterns of various fault statuses are observed. The variance in the patterns of the dynamic error trajectory diagrams of various statuses is used as characteristics, and the extension neural network theory is used to build the matter-element model.

## 3. Extension Neural Network Theory

The extension theory [18] aims to determine the regularity of statuses based on different statuses of things and extensibility, and concludes characteristics by mathematical operation. The extension theory is mainly divided into matter-element theory and extension set. The neural network is widely used in different areas, especially in control and diagnosis. The extension neural network [19] combines the above two theories. This system not only has the simple calculation of extension theory, but also has the ability of neural network to build non-linear neurons and strong adaptation. In comparison to other types of traditional neural network, the extension neural network has faster learning and higher accuracy, and it uses a few data to establish weights. Therefore, the extension neural network can use training data to build the matter-element model corresponding to the fault category, adjust and update the weights of various eigenvalues, and calculate the extension distance between the analyte and various categories, so as to identify the category of an analyte accurately.

**Figure 3.**
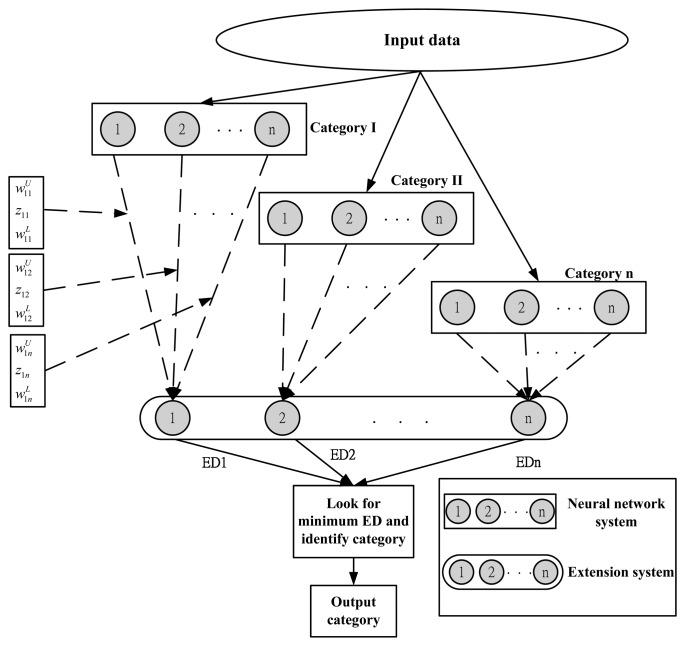
Extension neural network architecture.

Figure 3 is a schematic diagram of the extension neural network architecture. First, the data are classified and imported into the neurons of the input layer. The number of neurons of the input layer is determined by the characteristic number of the matter-elements to be identified. The output layer stores the calculated extension distances. The extension neural network has only two layers of neurons. One is the input layer, the other one is the output layer. There is no hidden layer of the traditional neural network. As the number of neurons of the hidden layer in the neural network is determined by expertise, whereas the extension neural network is free from the hidden layer, it is unnecessary to calculate the number of hidden neurons, so the fault recognition rate can be increased. In Figure 3, the dotted line connecting two layers indicates the weights, which include the upper bound of weight, weight center and lower bound of weight. Finally, the minimum extension distance is determined in the extension distance of various categories stored in the output layer, such as identifying the fault category. The extension neural network, like other neural networks, is divided into training stage and diagnostic identification stage.

General neural networks can be divided into two types. One is the supervised learning network, and the other one is the unsupervised learning network. In terms of supervised learning network, the known learning samples are imported into the neural network for recognition training. The learning is finished if the recognition results meet the known samples. On the other hand, if the recognition results do not meet the known samples, the weight is adjusted for the recognition results to meet the known samples. This step is repeated until all the recognition results meet the known samples. As the extension neural network is supervised learning, the accuracy of diagnostic system can be increased by adjusting the weight, and the error rate can be reduced, and the accuracy rate can be increased. The learning sample *X*={*X*^1^, *X*_2_, *X*_3_, …, *X_Np_*} is defined before the learning procedure, where *N_p_* is the total number of learning samples, and each sample contains data characteristics and category $X_{i}^{k}=\{X_{i1}^{k},X_{i2}^{k},X_{i3}^{k},\ldots ,X_{in}^{k}\}$, where the learning sample i=1,2,3,…,*N_p_*, *n* is the total number of characteristics in the matter-element model, *k* is the category of sample data. The extension distance is used to calculate the distance between sample and *k*-th cluster, the mathematical expression is:

(6) $$ED=\sum_{j=1}^{n}\left[\frac{\left|x_{ij}^{k}-z_{kj}\right|-\left(w_{kj}^{U}-w_{kj}^{L}\right)/2}{\left|\left(w_{kj}^{U}-w_{kj}^{L}\right)/2\right|}+1\right]$$

where $x_{ij}^{k}$ is the *j*-th characteristic of the *i*-th learning sample of category *k*, *z_kj_* is the weight center between the *j*-th input and the *k*-th output, $w_{kj}^{U}$ and $w_{kj}^{L}$ are the upper and lower limits of weights of the *j*-th input and the *k*-th output.

**Figure 4.**
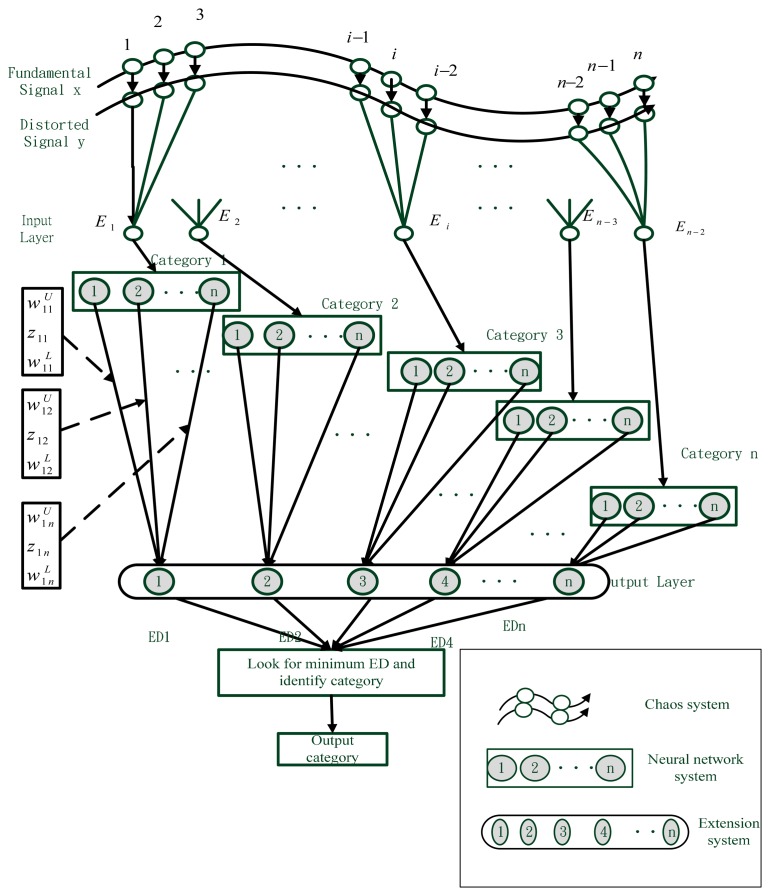
Chaos synchronization extension neural network architecture.

This study used the synchronization system as the basic detector for fault diagnosis. Figure 4 is the schematic diagram of the chaos synchronization neural network extension architecture. First, the basic signal of the normal solar array is included in the chaos synchronization master system, and the fault distorted signal is included in the slave system. Once the fault distorted signal is received, the chaos synchronization system tracks the error, and transfers the error signal to the neural network (input layer). The number of neurons of the input layer is determined by the characteristic number of the matter-elements to be identified. The output layer stores the calculated extension distance. Then the extension matter-element model can be used to classify the fault causes completely. The basic architecture is shown below.

## 4. Experimental Results

**Figure 5.**
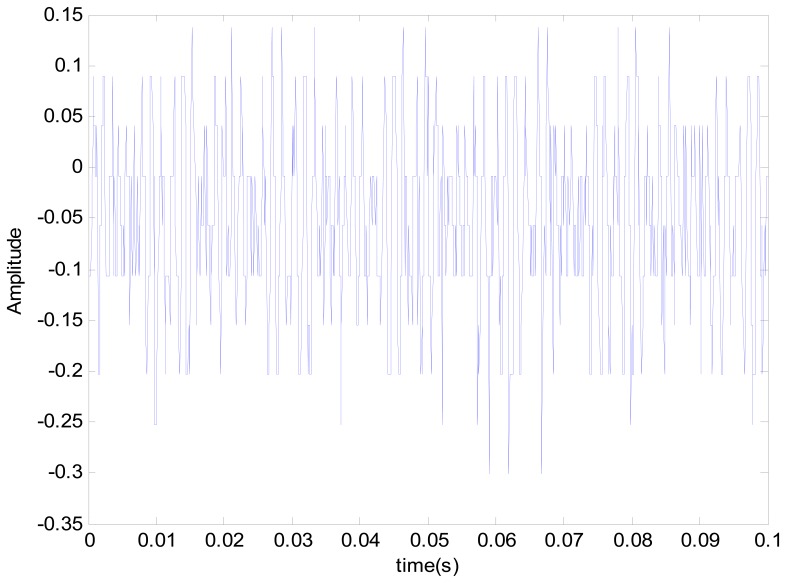
XXY positioning stage vibration signal.

**Figure 6.**
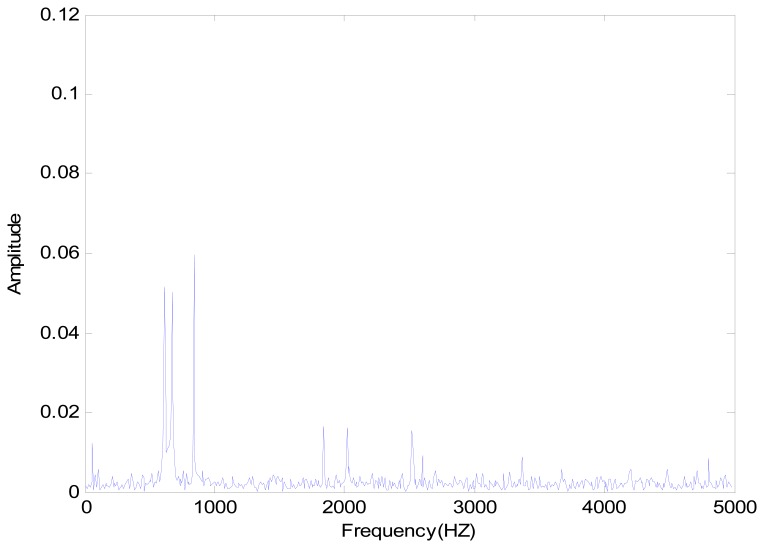
XXY positioning stage vibration signal spectrum.

The positioning stage used in this study is a XXY stage, developed and made by Chiuan Yan Technology (Changhua, Taiwan). A single accelerometer was used as the sensor. The dSPACE signal adapter plate transferred data to the computer. The signal changes were extracted by the filtering resulting from the vibration signals of various statuses. The extracted signals were imported into the master and slave chaotic systems. The differences between faults were increased by chaotic characteristics, so as to solve the difficulty in recognizing too small signals. The signal through the master and slave chaotic systems was more sensitive to the stage status. The dynamic error trajectory was drawn, and the pattern analysis was helpful to the observation of faults. Finally, the matter-element model of various fault statuses was built on the pattern characteristics of various statuses. The correlation function of the analytic stage for various fault matter-element models was calculated. The collision fault for this experiment was created by a sending pulse volume above the physical limit of the positioning stage. The positioning stage detection method proposed in this study could identify normal status and collision fault stage fault statuses. The collision faults were divided into Y-direction and X-direction. The vibration signal of stage and its FFT spectrum in normal status are shown in Figures 5 and 6. It can be seen that there were observed three prominent amplitudes in the spectrum. The experimental result showed the three prominent amplitudes are approximately the X1 signal at 550 HZ to 650 HZ, X2 signal at 650 HZ to 750 HZ, and Y signal at 800 HZ to 900 HZ.

**Figure 7.**
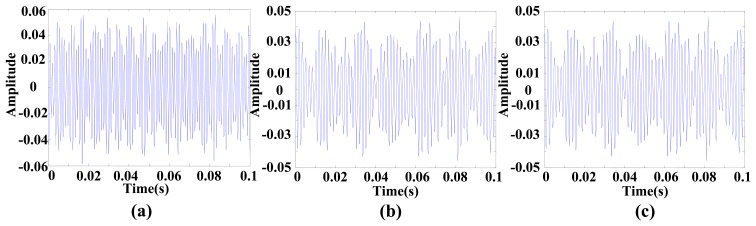
Characteristic signals: (**a**) Y signal; (**b**) X1 signal; (**c**) X2 signal.

**Figure 8.**
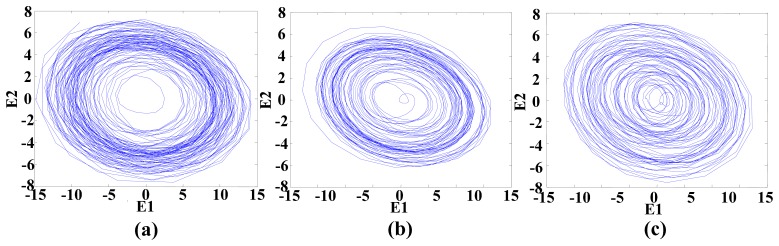
Dynamic error trajectories of the extracted signals: (**a**) Dynamic error trajectory of Y signal; (**b**) Dynamic error trajectory of X1 signal; (**c**) Dynamic error trajectory of X2 signal.

This vibration signal was extracted by bandpass filtering, and the characteristic signals Y, X1 and X2 were extracted. The characteristic signals can be observed in Figure 7. It can been seen that the vibratory magnitude of these extracted characteristic signals was not large, so that these characteristic signals were disadvantageous for fault diagnosis. The variation was very small when a fault occurred, so the Lorenz master and slave chaotic systems were used to strengthen the characteristics with the chaotic characteristics. The dynamic error resulted from the master-slave system was extracted. Finally, the dynamic errors *E*_1_ and *E*_2_ were selected for drawing the trajectory diagram. The dynamic error trajectories of the extracted signals are shown in Figure 8. It can be seen that the characteristic signal was affected by Lorenz chaos and the pattern characteristics were better than the original signals for identifying positioning stage faults.

**Figure 9.**
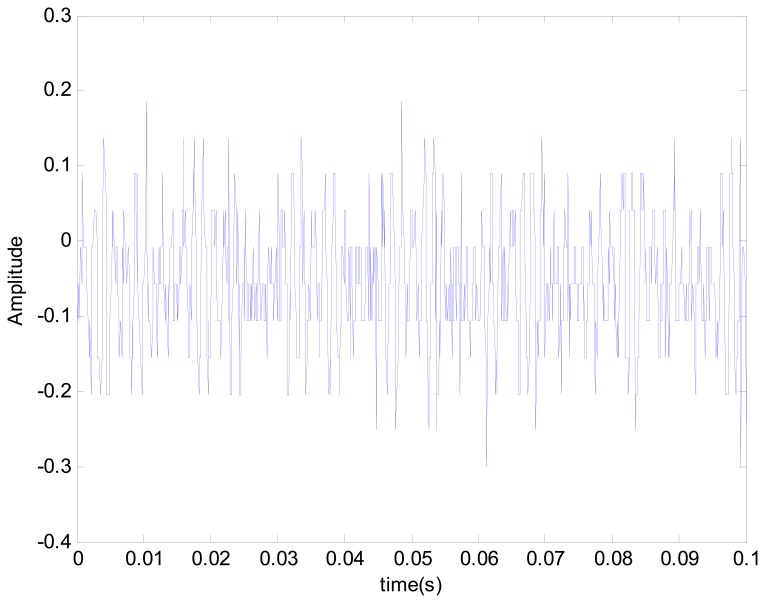
Y-direction stage collision fault vibration signal.

**Figure 10.**
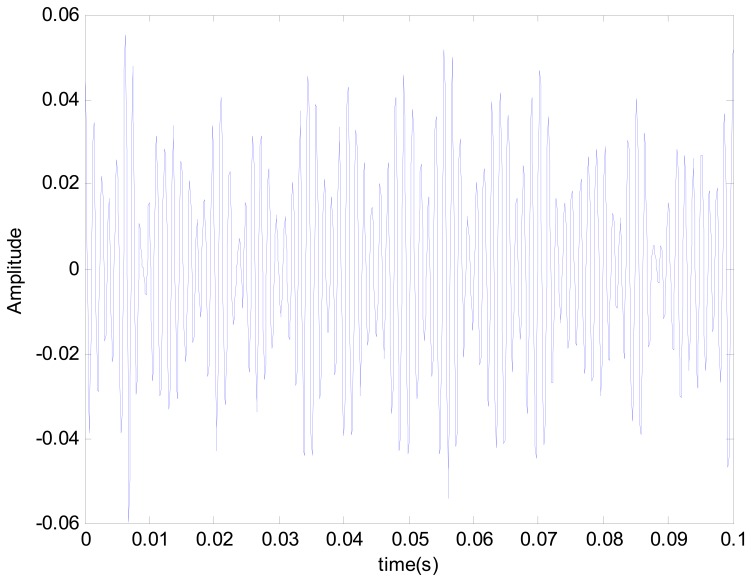
Y characteristic signal of Y-direction stage collision.

**Figure 11.**
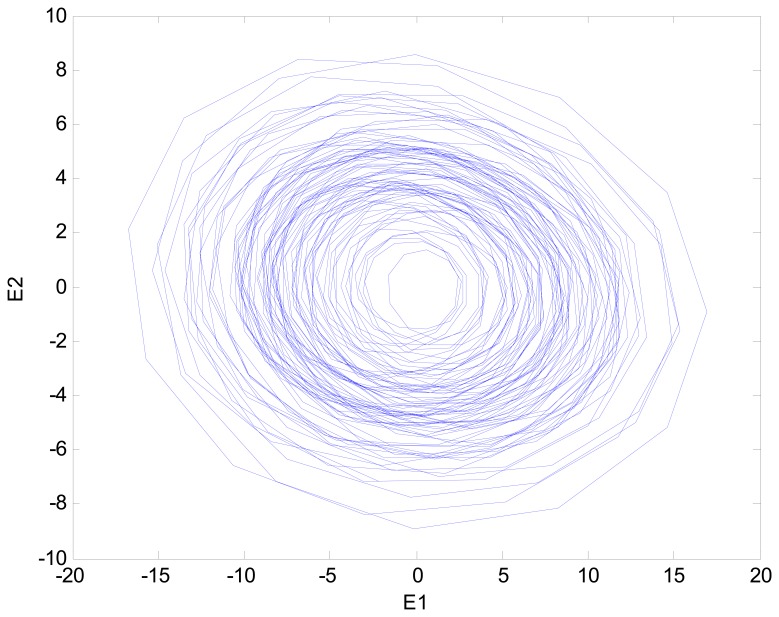
Y dynamic error trajectory of Y-direction stage collision.

**Figure 12.**
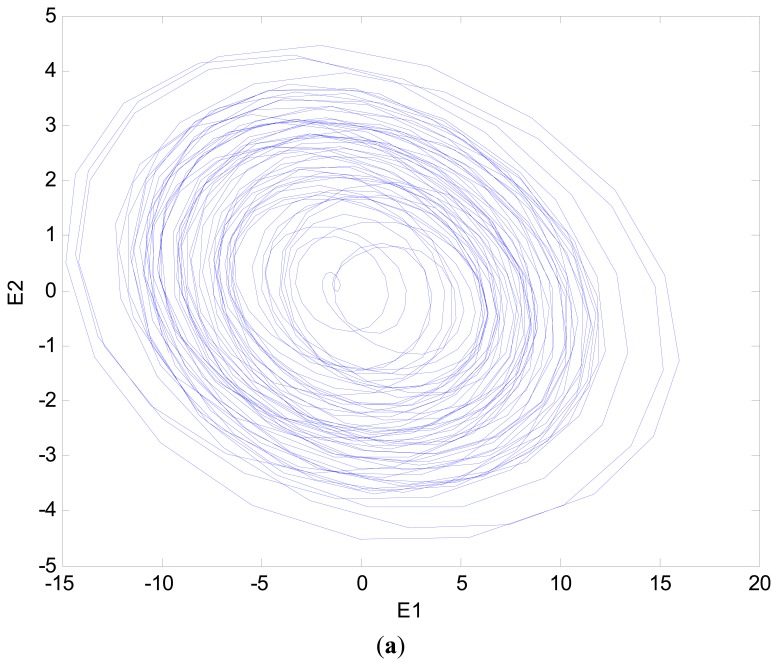

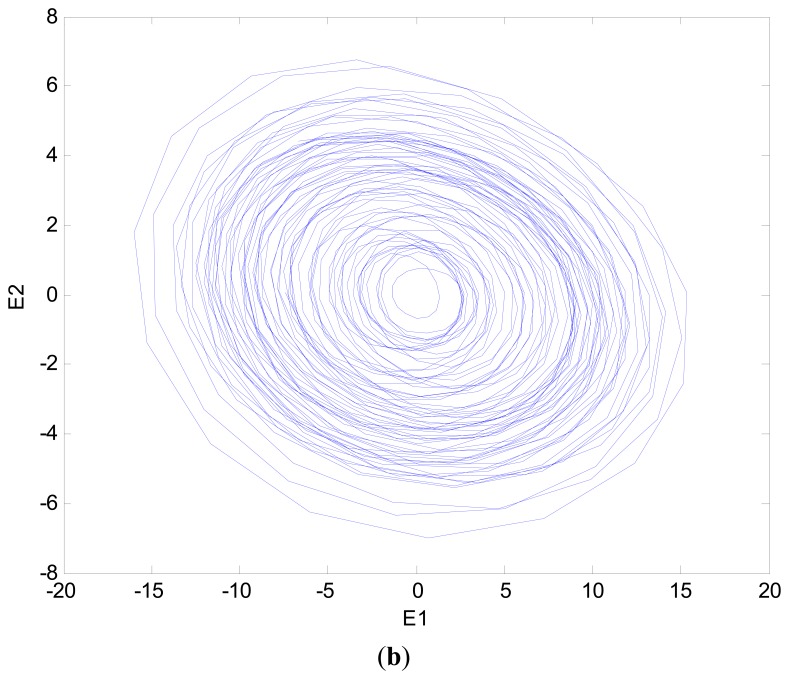
(**a**) X1 dynamic error trajectory of X-direction stage collision; (**b**) X2 dynamic error trajectory of X-direction stage collision.

When the positioning stage collided, different collision directions generate different vibratory magnitudes. The signals Y, X1 and X2 extracted by filtering thus changed. However, the change was very slight, so it was difficult to identify collisions. The fault status was identified easily by using master and slave chaotic systems. When the positioning stage had a Y-direction collision fault, the measured vibration signal changed. The vibration signal is shown in Figure 9. It was difficult to distinguish the collision fault status from the normal status in the vibration signal. This change was expected to be displayed by the Y characteristic signal. The Y characteristic signal is shown in Figure 10. The change in the Y characteristic signal only showed a slight increase in the amplitude, but the stage collision status was not identified. Therefore, the master and slave chaotic systems were used to observe the Y characteristic signal. The dynamic error trajectory is shown in Figure 11. In the dynamic error trajectory diagram derived from the Y characteristic signal, the pattern had obvious changes. When the positioning stage collided, in the trajectory diagram drawn from *E*_1_ and *E_2_*, the trajectory moved towards the pattern center <0,0>, so that the trajectory pattern shrank. When *E*_1_ was 0 as center point, the positive and negative centroids of *E*_1_ moved towards the center point, so that the distance between them was shortened. However, in the dynamic error trajectory diagram, the range of pattern was similar to the normal status, or the trajectory pattern expanded slightly. In other words, *E*_1_ maximum value and minimum value did not change, or only increased slightly. This variation in characteristic pattern also occurred in the dynamic error trajectory of X1 and X2 characteristic signals. However, in the Y-direction collide status, the variation was not as apparent as that in Y dynamic error trajectory. When the positioning stage had a Y-direction collision fault, the X1 and X2 dynamic error trajectories had the same pattern change, as shown in Figure 12. The occurrence and directionality of collision fault were found by the change in the characteristic pattern.

According to the dynamic error graph, the dynamic error *E*_1_ was selected as the major fault feature because its variation was more obvious than other dynamic errors'. The distance between positive and negative centers of gravity of *E*_1_ was used as the first feature. The maximum-minimum distance of *E*_1_ was the second feature, and the sum of maximum-minimum distances of *E*_1_ resulted from various characteristic signals was the third feature. The normal status and collision fault were identified from the changes in the three features. The matter-element models of various fault statuses were built by using the extension neural network based on the changes in the three features. The fault matter-element models and joint domain are shown in Table 1, where *C*_1_, *C*_2_ and *C*_3_ denote the distance between positive and negative centers of gravity of *E*_1_ of Y, X1 and X2, respectively, *C*_4_, *C*_5_ and *C*_6_ denote the distance between maximum value and minimum value of *E*_1_ of Y, X1 and X2, respectively, *C*_7_ is the aggregation of *C*_4_, *C*_5_ and *C*_6_. Finally, the correlation function of various matter-element models was calculated, in order to find out the fault matter-element model closest to the present stage.

The positioning stage collision fault matter-element model was used for stage diagnosis. The signal adapter panel imported the stage vibration signal into the computer. The signal input was extracted by ADC (analog-to-digital converter), and the extraction frequency of ADC was 10 KHz. The stage status was updated every 0.5 s, so as to check whether the positioning stage has a collision fault instantly. In terms of fault detection, there were 10,000 fault status data points in the test. The diagnostic result of collision fault is shown in Table 2. The accuracy rate of collision diagnosis was lower than normal diagnosis because the collision signal was too similar to the normal signal. The result was not ideal, but the detection had a certain effect.

## 5. Conclusions

This study focused on XXY stage collision fault detection. The characteristic signal was imported into master and slave chaotic systems to extract the dynamic error resulting from the master and slave chaotic systems. As the signal changed violently due to chaos phenomena, the features of various fault statuses were increased, and the pattern features were extracted from the dynamic error trajectory. Finally, the matter-element model was built by using an extension neural network. The present fault status can be known by calculating the degree of correlation of the analysis. In comparison to other identification methods, the extension neural network retains the simplicity of extension operation and the adaptability of a neural network. The combination of master-slave chaos error extraction system and extension neural network theory can identify faults rapidly. The proposed system only requires one sensor for detection, thus lowering the cost.

## Figures and Tables

**Table 1.** Matter-element model of XXY stage fault status.

$\text{Stage status}\;1=\left[\begin{array}{ccc} \text{normal} & C_{1} & <11,13>\\ & C_{2} & <11,13>\\ & C_{3} & <11,13>\\ & C_{4} & <25,35>\\ & C_{5} & <25,35>\\ & C_{6} & <30,40>\\ & C_{7} & <95,110> \end{array}\right]$

$\text{Stage status}\;2=\left[\begin{array}{ccc} Y\_\text{Collide} & C_{1} & <9,11>\\ & C_{2} & <11,12>\\ & C_{3} & <11,13>\\ & C_{4} & <25,35>\\ & C_{5} & <25,35>\\ & C_{6} & <35,45>\\ & C_{7} & <100,120> \end{array}\right]$

$\text{Stage status}\;3=\left[\begin{array}{ccc} X\_\text{Collide} & C_{1} & <11,13>\\ & C_{2} & <13,15>\\ & C_{3} & <8,10>\\ & C_{4} & <25,35>\\ & C_{5} & <35,40>\\ & C_{6} & <25,35>\\ & C_{7} & <100,120> \end{array}\right]$

$\text{Joint domain}=\left[\begin{array}{ccc} {\text{E}}_{1} & C_{1} & <3,13>\\ & C_{2} & <4\text{.5},15>\\ & C_{3} & <7,13>\\ & C_{4} & <10,35>\\ & C_{5} & <25,40>\\ & C_{6} & <25,45>\\ & C_{7} & <70,120> \end{array}\right]$

**Table 2.** XXY stage collision fault accuracy rate.

**Fault Status**	**Accuracy**
Normal	97%
Y Collide	75%
X Collide	76%
